# Unlocking prevention: the role of health literacy in cervical cancer screening: community nursing perspective

**DOI:** 10.1186/s12912-025-02797-4

**Published:** 2025-02-11

**Authors:** Shaimaa Mohamed Amin, Mona Metwally El-Sayed, Ahmed Hashem El-Monshed, Mohamed Hussein Ramadan Atta

**Affiliations:** 1 Lecturer of Community Health Nursing, Faculty of Nursing, Damanhour University, Damanhour City, Egypt; 2 Psychiatric and Mental Health Nursing department, Faculty of nursing, Alexandria University, Alexandria City, Egypt; 3 Department of Nursing, College of Health and Sport Sciences, University of Bahrain, Manama City, Bahrain; 4 Department of Psychiatric and Mental Health Nursing, Faculty of Nursing, Mansoura University, Mansoura, Egypt; 5 Psychiatric and mental health nursing department, Faculty of nursing, Alexandria University, Alexandria City, Egypt

**Keywords:** Cervical Cancer, Community nursing, Health literacy, Screening behavior

## Abstract

**Introduction:**

Cervical cancer presents a significant global public health challenge, particularly affecting low- and middle-income countries like Egypt. Despite the availability of effective screening methods such as Pap smears and HPV testing, the incidence of cervical cancer remains high in Egypt. Health literacy, which refers to the ability to access, understand, and utilize basic health information and services to make informed decisions, is crucial in influencing individuals’ health behaviors, including their participation in cancer screening programs.

**Objectives:**

To examine the correlation between health literacy levels and cervical cancer screening behaviors among women.

**Methods:**

This study employed a multi-site cross-sectional research design from September 2023 to January 2024. The research was conducted at four primary health care (PHC) facilities in the Damanhur district of Egypt. Three hundred fifty women participated in the study, completing a comprehensive questionnaire that included a Woman’s Social and Health Form, a Cervical Cancer Knowledge Scale, a Cervical Cancer Screening Behaviors Scale, and a Health Literacy Scale (HLS-SF12).

**Results:**

The study revealed significant relationships between the importance of health literacy (HL) in understanding cervical cancer (CC) knowledge and screening behaviors among Egyptian women. A positive correlation was found between Knowledge and HL (*r* = 0.507, *p* < 0.001). Conversely, perceived barriers negatively correlated with knowledge and HL (*r* = -0.172, *p* < 0.05; *r* = -0.277, *p* < 0.01). The regression analysis revealed that higher levels of HL were significantly associated with greater knowledge about CC (B = 0.148, *p* < 0.001). Conversely, knowledge about CC was also found to be a strong predictor of higher HL levels (B = 1.205, *p* < 0.001). These results highlight the bidirectional relationship between HL and knowledge, where improvements in one can enhance the other.

**Conclusion:**

Addressing misconceptions and increasing knowledge about the importance of regular screenings, mainly through accessible and culturally appropriate channels, could lead to an improved uptake of cervical cancer screening services. Overall, this study lays a foundation for future research to continue exploring ways to improve cervical cancer prevention and control efforts among women.

**Clinical trial number:**

Not applicable.

## Introduction

Cervical cancer (CC) presents a significant global public health challenge, particularly impacting middle-aged women in low- and middle-income countries [1]. In 2018, there were approximately 570,000 new cases of CC worldwide, accounting for 6.6% of all female cancer diagnoses, with over 85% of CC-related deaths occurring in these countries [2, 3]. In Egypt, CC is the 14th most common cancer among females of all age groups, with an estimated 33.2 million women at risk. In 2020, there were 1320 reported cases, making CC the 11th most common cancer among women aged 15 to 44 years [4]. It is also the 12th leading cause of cancer-related deaths among women aged 15 to 44 years in Egypt, with an age-standardized mortality rate of 1.5 per 100,000 individuals per year [2]. In 2018, Egypt reported 969 new cases of CC, resulting in 631 deaths, representing 2% and 1.6% of all cancers and cancer-related deaths among women, respectively [1, 2].

However, despite the availability of screening services, the participation rates among women are suboptimal. Health literacy, the ability to acquire, process, and comprehend basic health information and services necessary for appropriate health decisions, is a critical factor contributing to this disparity [2, 4]. Health literacy is pivotal in preventive healthcare as it empowers individuals to comprehend the importance of screening, navigate healthcare systems, and adhere to recommended health practices. Community nurses play a vital role in public health by providing education, support, and interventions to improve health outcomes. Given their close interaction with the community, they are ideally positioned to address barriers to cervical cancer screening and enhance health literacy among women.

The World Health Organization (WHO) has identified human papillomavirus (HPV) infection as the primary cause of cervical cancer (CC). Additional risk factors include early sexual activity, multiple sexual partners, hormonal influences, genetics, smoking, high parity, and lower socioeconomic status [5–8]. Cervical cancer is preventable, according to several research studies [3, 5, 9]. The progression from precancerous lesions to invasive CC typically takes up to two decades. CC is preventable through measures such as HPV vaccination and effective screening, which can significantly reduce incidence and mortality [2, 10]. Global initiatives advocate for organized screening programs, highlighted in the WHO’s 2020 World Cancer Report. Research indicates that offering HPV vaccination to all women and implementing high-quality cytological screening programs could prevent up to 90% of HPV-associated CC cases [2, 11].

The accessibility of Pap tests and other screening methods like VIA (Visual Inspection with Acetic Acid) or HPV (Human Papillomavirus) testing in Egypt can vary depending on geographic location, healthcare infrastructure, and available resources. While Pap tests may be available in certain healthcare facilities in Egypt, their accessibility could be limited, especially in rural or underserved areas. However, efforts may be underway to improve access to cervical cancer screening services, including Pap tests. Regarding VIA and HPV testing, their availability and usage in Egypt may be increasing, particularly as technology advances and awareness about cervical cancer prevention grows. VIA is a relatively simple and cost-effective screening method that can be performed by trained healthcare providers, making it potentially more accessible in resource-limited settings compared to Pap tests. HPV testing, which detects the presence of high-risk HPV strains associated with cervical cancer, is becoming increasingly important in cervical cancer screening programs globally. Its availability in Egypt may depend on healthcare infrastructure and affordability [12].

Nevertheless, the disparity in CCS and HPV vaccination rates is evident, with approximately 20% of women in low- and lower-middle-income countries (LMICs) having undergone CCS, compared to the 60% reported in high-income countries [12, 13]. Currently, Egypt lacks a national screening program for CC, with Papanicolaou (PAP) smear screening considered potentially cost-ineffective, given the comparatively low rates of CCS among Egyptian women when compared to Western countries [14]. The Egypt Health Issues Survey (2015) reported that only 0.3% of Egyptian women had undergone a Pap Smear [15]. This indicates a suboptimal uptake of CCS in the country. Several factors contribute to this situation, including socioeconomic disparities, where limited access to healthcare services affects screening participation in rural areas. Additionally, inadequate health literacy levels have been identified as a significant barrier, impacting the understanding of the importance of early detection and preventive measures [16–18].

Health literacy (HL) refers to individuals’ ability to access, understand, and apply basic health information, crucial for making informed decisions about their health [19]. Recognized as a critical social determinant of health, HL ensures access to quality, patient-centered care [20]. Lower HL levels are associated with adverse health outcomes, such as increased hospitalizations, medication non-adherence, and reduced participation in preventive measures like cancer screening programs [21]. By correlating health literacy levels with health beliefs, the study aims to elucidate how individuals’ abilities to comprehend and process health information may influence their perceptions of screening efficacy, necessity, and potential barriers. This exploration is essential as it allows a deeper understanding of the cognitive processes driving screening decision-making. Studies consistently demonstrate a strong correlation between HL and participation in cancer screening, with evidence indicating that individuals with higher HL are more likely to adhere to screening recommendations [22–23]. Addressing HL is thus essential for promoting proactive health behaviors [24].

Community health nurses are at the forefront of the battle against CC. Their roles encompass raising awareness, educating the public, and enhancing access to crucial screening and vaccination initiatives. These healthcare professionals engage closely with individuals, families, and communities to advocate for preventive measures and early detection. They distribute pertinent information and guide individuals toward further assessment and treatment when necessary. By collaborating with other healthcare providers, community health nurses significantly enhance the effectiveness of prevention strategies and vaccination programs [25, 26].

This research delves into the critical influence of health literacy on cervical cancer screening (CCS) behaviors, particularly in the absence of formal screening programs. By examining the correlation between health literacy and screening behaviors among Egyptian women, the study underscores the importance of addressing health literacy as a modifiable factor to enhance screening uptake. It highlights the need for integrating health literacy interventions into cervical cancer prevention strategies and emphasizes healthcare providers’ personalized education and communication strategies to increase screening rates. Moreover, the study calls for future research to explore innovative approaches to cervical cancer prevention in settings without established national programs, emphasizing health literacy’s crucial role in shaping screening behaviors. Overall, by elucidating the cognitive factors driving screening decision-making and addressing misconceptions, the study aims to inspire more women to prioritize CCS. Consequently, our study explores this issue to ascertain the correlation between CCS behaviors and HL among Egyptian women.

### Objectives

Determining the Role of Health Literacy in Cervical Cancer Screening.

### Research questions

- What is the level of knowledge about cervical cancer screening among the target population?
- What are the key predictors of knowledge regarding cervical cancer screening from a community nursing perspective?
- How does health literacy influence knowledge and engagement in cervical cancer screening practices?

## Method

### Research design

A cross-sectional research design involving multiple sites was conducted, adhering to the Strengthening the Reporting of Observational Studies in Epidemiology (STROBE) checklist.

### Setting

The study was conducted at four primary health care (PHC) facilities in Damanhur district, Egypt. Specifically, two rural PHC facilities, namely Shrouq Family Medicine Unit and Hamour Family Medicine Unit, and two urban PHC facilities, namely El-Helal Maternal and Child Health Center and Naser Family Medicine Center.

### Population, sample size

The requisite sample size was meticulously determined through a comprehensive power analysis utilizing G*Power version 3.1.9.7 [27]. This analysis, conducted at a 98% confidence level with a 5% alpha (significance) level, incorporated a suggested medium effect size of 0.15 as per Cohen’s (2013) guidelines [28]. Given the intricate nature of our regression analysis, which encompasses 27 predictors spanning the Cervical Cancer Knowledge Scale, HL Scale, Perceived Susceptibility and Severity, Perceived Benefits, Perceived Barriers, Cues to Action, and 21 demographic and clinical variables, the calculated minimum sample size stood at 289 individuals.

To proactively address potential challenges inherent in our chosen data collection methodology and accommodate an expected dropout rate of 30% [29], we have judiciously added 87 participants. This prudent adjustment bolsters our findings’ robustness, integrity, and reliability. Furthermore, while 376 women were sought to participate in and complete the survey, the final sample size for the current study, comprising participants who accepted and filled in the questionnaire, was 350.

The inclusion criteria for women participating in this study were carefully defined to ensure the relevance and accuracy of the findings. Eligible participants were required to be women in their 20s and 50s, at a stage in life where they are likely to be more actively engaged in health-related behaviors and decisions, including cervical cancer screening. This age range also aligns with the typical age range for cervical cancer screening recommendations, ensuring that the findings apply to women most likely to benefit from the study outcomes. Additionally, proficiency in Arabic was necessary to effectively understand and respond to the survey questionnaire. A fundamental requirement for inclusion was the expressed willingness of participants to partake in the study, demonstrating their informed consent to contribute to research efforts. Furthermore, participants needed to be available during the designated data collection period to complete the survey, ensuring the feasibility of their participation. The exclusion criteria for women participating in this study included a history of cervical cancer. This exclusion was implemented to ensure that the study focused on women without a current or past diagnosis of cervical cancer, thereby maintaining a homogenous sample for more accurate analysis. These carefully delineated inclusion criteria aimed to establish a sample of women meeting the study’s objectives, facilitating a comprehensive exploration of factors influencing CCS behaviors within this population subgroup.

**Fig. 1:**
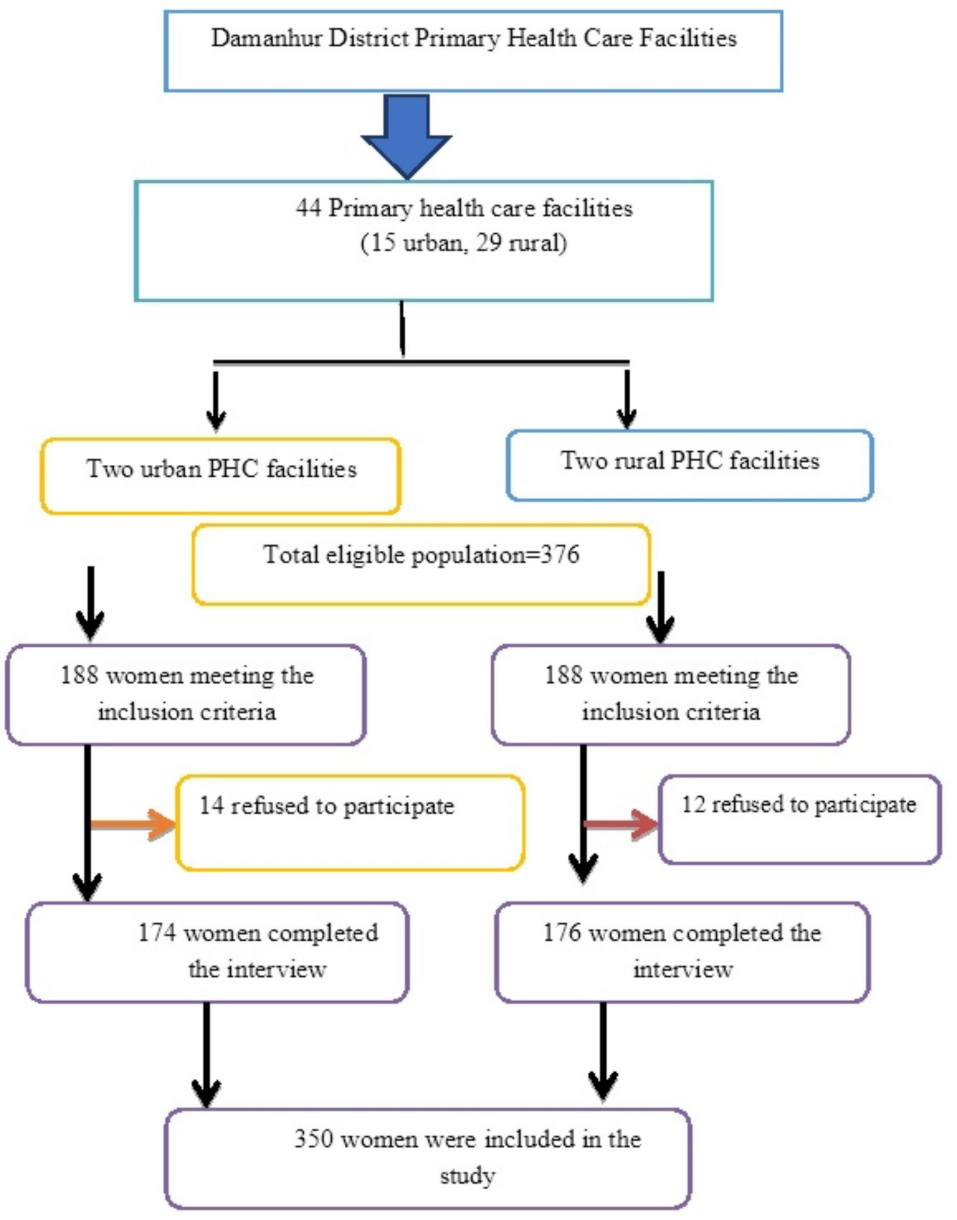
Participant’s recruitment flow chart

This study utilized a two-stage random sampling technique for participant recruitment. In the first phase, two urban and two rural Primary Health Care (PHC) facilities were selected from 44 such facilities in Damanhur district. In the second phase, a sample of 376 women was assembled using a systematic random sampling approach. This involved including every fourth woman arriving at the center/unit. The final sample size was 350 participants (Fig. 1).

### Measurements of interest

#### Woman’s social and health form

This form encompasses various dimensions crucial for understanding women’s health profiles. It includes socio-demographic data such as age, education level, marital status, residence, family type, employment status, and income. Additionally, it delves into medical history, including chronic diseases and family history of CC. Lifestyle factors were also evaluated, covering exercise habits, dietary regimen, sleep patterns, smoking behavior, exposure to passive smoking, periodic medical checkups, and radiation exposure. Finally, the form assesses CCS practices, including awareness, sources of information, screening history, frequency, recency, and HPV vaccination status. These comprehensive data points provide essential insights into women’s social contexts, health statuses, and preventive healthcare practices, aiding in tailored interventions and healthcare planning.

### Cervical cancer knowledge scale

Haward et al. (2022) developed and validated the Cervical Cancer Knowledge Scale as an instrument designed to assess women’s understanding of CC risk factors, symptoms, and preventive measures [30]. Comprising eight items, the scale prompts participants to indicate their knowledge regarding various aspects of CC by responding with “yes,” “no.“, and “don’t know.” The correct answer took (1), and the wrong /don’t know took (0). The assessment method relies on the total count of accurate responses, where a score exceeding 4 out of 8 signifies a satisfactory level of understanding regarding CC. This evaluation tool has undergone validation procedures and exhibited favorable internal reliability, as evidenced by Cronbach’s alpha coefficient of 0.76. The scale exhibits adequate fit statistics for both one-factor and two-factor models, suggesting its suitability for assessing CC knowledge in diverse populations [30].

### Cervical cancer screening behaviors scale

The Cervical Cancer Screening Behaviors Scale is a structured questionnaire designed to evaluate individuals’ attitudes and perceptions towards CCS, grounded in the Health Belief Model (HBM) [31]. The scale consists of 27 items, each rated on a 5-point Likert scale ranging from 1 (strongly disagree) to 5 (strongly agree). Total scores on the scale range from 27 to 135, with higher scores indicating a more positive perception of the benefits of engaging in cervical cancer screening. For example, items 1–8 assess perceived susceptibility and severity of CC, such as beliefs about personal risk and the impact of cancer on one’s life and family. Items 9–13 evaluate the perceived benefits of screening, such as mortality reduction, early detection, peace of mind, and health management. Items 14–20 assess perceived barriers to screening, including time constraints, cost, lack of knowledge about screening locations, forgetfulness, embarrassment, and prioritization of other health concerns. Finally, items 21–27 capture cues to action, such as recommendations from friends, family, and healthcare providers. Cui et al. (2022) conducted a factor analysis on the 27 variables, revealing that all variables could be classified into seven factors consistent with the HBM framework [31]. Additionally, internal consistency among the items within each scale was assessed using Cronbach’s alpha coefficient. The coefficient values were 0.818 for “seriousness of cancer,” 0.820 for “benefits of cancer screening,” 0.872 for “the importance of cancer screening,” 0.681 for “cues to participation in screening,” 0.699 for “cancer susceptibility,” 0.812 for “barriers to participation at the time of cancer screening,” and 0.752 for “barriers to participation before cancer screening.” Generally, a Cronbach’s alpha coefficient exceeding 0.748 indicated satisfactory internal consistency for the variables representing each factor [32].

### Health literacy scale (HLS-SF12)

Health Literacy Scale comprises twelve items assessing individuals’ proficiency in various health-related tasks [33], drawing on prior work by Sørensen et al. (2012) using the HLS-EU-Q47. Participants rate the perceived difficulty of each task on a 4-point Likert scale ranging from “very difficult” to “very easy“ [18]. Health Literacy Scale scores ranged from 0 to 50, where higher scores represented greater health literacy. Scores of 33 and below indicated limited literacy. Tasks include locating information on illness treatment, understanding medical emergencies, assessing treatment options, adhering to medication instructions, managing mental health issues, comprehending the necessity of health Screenings, evaluating media health information reliability, making health-related decisions based on advice, accessing the information on health attitudes and nutrition, understanding food packaging information, identifying behaviors related to health, and making decisions to enhance personal health. The HLS-SF12 has been shown to possess satisfactory psychometric properties, including solid reliability, with a Cronbach’s alpha coefficient of 0.85. It also demonstrates good criterion-related validity and moderate to high levels of item-scale convergent validity [32].

### Ethical considerations

Approval from the Research Ethics Committee (REC) at the College of Nursing, Damanhur University, was obtained on July 22nd, 2022, with the approval code number (52-d). Participants were informed of the study’s objectives, and their data were assured to be used exclusively for research purposes. Additionally, participants were informed of their right to refuse participation or withdraw from the study at any point without facing adverse consequences. Informed written consent was obtained from women who agreed to participate. Anonymity was preserved, and data confidentiality was maintained throughout the study.

### Data collection

The research obtained formal approval to commence from the relevant authorities at the colleges of nursing. The three study’s tools were translated into Arabic using a standardized process that included forward translation by a bilingual expert and back-translation into English by another independent bilingual expert. This approach ensured linguistic and conceptual equivalence with the original tool. To validate the appropriateness of the translated version, face validity was conducted with a panel of experts in nursing and public health, who reviewed the tool to ensure clarity, relevance, and cultural appropriateness. The three tools utilized in this study were confirmed to be valid and reliable for assessing cervical cancer knowledge, health literacy, and screening behaviors among the study population. The reliability of each tool was established using Cronbach’s alpha test, with values (0.78, 0.83, and 0.79, respectively), indicating good internal consistency.

A pilot study comprising 25 women not involved in the primary survey assessed the research instruments’ appropriateness, comprehensibility, and practicality. The pilot research findings revealed that no adjustments or alterations were required. Individuals who engaged in the pilot and reliability phases were omitted from the primary study population.

Recruiting community nurses for research by establishing partnerships with local healthcare facilities, such as clinics and community health centers, can provide access to a pool of qualified nurses who are already embedded in the community. These nurses can serve as key liaisons between the research team and the community, helping to facilitate participant recruitment and data collection.

Secondly, conducting informational sessions or workshops for nurses about the importance of the research and its potential impact on improving cervical cancer screening behaviors can help generate interest and engagement. These sessions can also provide training on research protocols and data collection methods to ensure that nurses are equipped with the necessary skills to carry out their roles in the study effectively.

Face-to-face interviews were conducted during the data collection process, and data collectors approached eligible women, clearly explaining the study’s purpose and obtaining informed consent before proceeding. Interviews are conducted in private settings to foster openness and honesty in responses. Quality control measures are rigorously implemented throughout data collection to ensure accuracy and completeness. This includes periodic review of completed questionnaires for consistency and immediate resolution of discrepancies. Furthermore, the gathering of data occurred during the period spanning from September 2023 to January 2024. Statistical significance was set at p < 0.05 for results to be considered significant, with more robust evidence of significance noted for p < 0.001 [33].”

### Statistical analysis

Data analysis was conducted using the Statistical Package for the Social Sciences (SPSS), version 29. Categorical data were summarized as percentages and frequencies, while continuous variables were presented as Mean ± Standard Deviation (SD). The Pearson correlation test was employed to examine correlations among continuous parametric variables. Linear regression analysis was utilized to predict the dependent variable’s value based on the independent variables’ values.

## Results

Table 1 presents a comprehensive overview of the socio-demographic characteristics of the study participants. The distribution of participants across different age groups indicates a diverse sample, with 31.1% aged 20 to below 30, 40.9% aged 30 to below 40, and 28.0% aged 40 to 50 or above. Most participants, 71.1%, have attained a university education or higher. Regarding marital status, a significant portion of the sample is married (64.3%). Residence patterns show that 72.3% of participants reside in urban areas, while 27.7% live in rural settings. Family structures vary, with the majority (85.4%) being nuclear families and 14.6% representing extended families. Employment status is predominantly characterized by working individuals (82.6%).

**Table 1:** Socio-demographic characteristics of the study participants (*N* = 350)

Socio-demographic Factors		*N*	*%*
Age in Years	20–29	109	31.1
	30 - 39	143	40.9
	40–50	98	28.0
Level of Education	Basic	6	1.7
	Secondary	95	27.1
	University or Higher	249	71.1
Marital Status	Single	84	24.0
	Married	225	64.3
	Widow	15	4.3
	Divorced	26	7.4
Residence	Urban	253	72.3
	Rural	97	27.7
Family Type	Nuclear	299	85.4
	Extended	51	14.6
Employment Status	Not Working	61	17.4
	Working	289	82.6
Family Income	Enough	74	21.1
	Enough & save	36	10.3
	Not Enough	240	68.6
History of Chronic Disease	Yes	62	17.7
	No	288	82.3
Family History of CC	Yes	20	5.7
	No	330	94.3
Age at Marriage in Years	> 20	96	27.4
	≥ 20	170	48.6

Table 2 provides insights into the lifestyle behaviors of the study participants. A relatively low percentage of participants engage in regular exercise, with only 4.9% reporting positively. Regular exercise was defined as engaging in physical activity for at least 150 min per week, based on international guidelines for moderate-intensity exercise. Dietary habits reveal a nearly equal split, with 46.3% adopting a healthy nutritional regimen and 53.7% leaning towards unhealthy choices such as fast foods. Sleeping patterns are predominantly characterized by insomnia, with 67.4% of participants reporting irregular sleep. Regular sleep was defined as maintaining consistent sleep and wake times, with a sleep duration of 6–8 h per night. Medical checkup practices are infrequent, with only 5 participants (1.4%) reporting regular checkups.

**Table 2:** Lifestyle behaviors of the study participants (*N* = 350)

Lifestyle		*N*	*%*
Regular Exercise	Yes	17	4.9
	No	333	95.1
Dietary Regimen	Healthy	162	46.3
	Unhealthy (fast foods)	188	53.7
Sleeping Pattern	Regular	114	32.6
	Insomnia	236	67.4
Smoking Condition	Smoker	4	1.1
	Non-Smoker	346	98.9
Passive Smoking	Yes	204	58.3
	No	146	41.7
Medical checkup	Yes	5	1.4
	No	345	98.6
Exposure to Radiation	Yes	10	2.9
	No	340	97.1

Table 3 outlines the cervical cancer screening practices among the study participants. For those who have, the sources of information vary, with 2.3% citing family and friends, 12.3% obtaining information from social media, 1.7% from radio or television, 11.1% from healthcare providers, and 2% from other sources. Concerning the actual practice of CCS, only 5 participants (1.4%) report having undergone cervical cancer screening, while the vast majority (98.6%) have not. Among those screened, 1.1% had their last screening one year ago. When considering only eligible participants aged 30 and above, the percentage of those who have undergone screening could be much higher, with only 2.07% of the eligible group reporting screening. This highlights a significant gap in cervical cancer screening practices within this population. Regarding the reception of the Human Papillomavirus (HPV) vaccine, only two participants reported having received it.

**Table 3:** Cervical Cancer Screening practices among the study participants (*N* = 350)

Cervical Cancer Screening Practices		*N*	*%*
Source of Knowledge	Family and Friends	8	2.3
	Social media	43	12.3
	Radio or Television	6	1.7
	Health Care Providers	39	11.1
	Other	7	2
Performing CCS	Yes	5	1.4
	No	345	98.6
Time of last CCS	One year ago	4	1.1
	More than one year	1	0.3
	No Screening	345	98.6
Receiving HPV	Yes	2	0.6
	No	348	99.4

In Table 4, the study participants demonstrated varying levels of awareness regarding cervical cancer. Notably, 40.3% recognized the lower risk associated with smoking, while 42.9% identified the higher risk linked to multiple sexual partners. Symptoms like vaginal bleeding between periods (50.6%), persistent unpleasant discharge (44.3%), and discomfort during sex (53.7%) were reasonably well-acknowledged. Additionally, awareness of signs such as bleeding after menopause (55.4%) and after sex (53.4%) was notable. However, despite a majority understanding (52.6%) that Pap tests can detect cervical abnormalities before cancer develops, there remains room for enhancing knowledge.

**Table 4:** Cervical Cancer knowledge as reported by the study participants (*N* = 350)

Items	Correct Answers	
	*N*	%
A woman is at lower risk for developing cervical cancer if she smokes	141	40.3
A woman is at higher risk of developing cervical cancer if she has had more than five sexual partners in her lifetime	150	42.9
Vaginal bleeding between periods can be a sign of cervical cancer	177	50.6
Persistent vaginal discharge that smells unpleasant can be a sign of cervical cancer	155	44.3
Discomfort or pain during sex can be a sign of cervical cancer	188	53.7
Vaginal bleeding after menopause can be a sign of cervical cancer	194	55.4
Vaginal bleeding during or after sex can be a sign of cervical cancer	187	53.4
The Pap test can detect abnormal cells of the cervix before they become cancer	184	52.6

Table 5 presents the descriptive statistics for the study measures. Knowledge (M = 3.93, SD = 2.93) ranged from 0 to 8, reflecting diverse levels of understanding. Health Literacy (M = 25.39, SD = 8.91) demonstrated moderate variability, with scores ranging from 12 to 48. Perceived Susceptibility and Severity (M = 20.82, SD = 4.96) varied from 8 to 40, indicating differing risk and severity perceptions. Perceived Benefits (M = 11.57, SD = 3.85) showed variability in how participants viewed the benefits of the behavior, with scores ranging from 4 to 20. Perceived Barriers (M = 30.30, SD = 9.95) indicated moderate to high perceived obstacles, ranging from 11 to 55. Lastly, Cues to Action (M = 10.28, SD = 3.39) ranged from 4 to 20, reflecting varying responses to prompts for health behavior.

**Table 5:** Means, Standard Deviations, reliability measures, and correlation among the study variables

Study Measures	M	SD	Min	Max	Knowledge	HL	Perceived Susceptibility & Severity	Perceived Benefits	Perceived Barriers	Cues to Action
Knowledge	3.93	2.926	0	8	1					
HL	25.39	8.911	12	48	0.507**	1				
Perceived Susceptibility & Severity	20.82	4.955	8	40	0.219**	0.290**	1			
Perceived Benefits	11.57	3.846	4	20	0.291**	0.416**	0.529***	1		
Perceived Barriers	30.30	9.950	11	55	-0.172**	-0.277**	-0.014	-0.104	1	
Cues to Action	10.28	3.393	4	20	0.032	0.081	0.240**	0.222**	0.035	1

HL: Health Literacy; Scores are mean (M) and Standard Deviation (SD) values, *r =* Pearson correlation

**. Correlation is significant at the 0.01 level (2-tailed)

Additionally, the correlation matrix in this table reveals significant relationships among crucial study variables. A positive and significant correlation between Knowledge and Health Literacy (*r* = 0.507, *p* < 0.001) indicates a strong association between factual understanding and broader health-related literacy. Conversely, perceived barriers exhibit negative correlations with knowledge and health literacy (*r* = -0.172, *p* < 0.05; *r* = -0.277, *p* < 0.01), indicating that higher perceived barriers are associated with lower knowledge and health literacy.

Table 6 shows the regression coefficients for Health Literacy and Screening Behaviors as predictors of Knowledge about Cervical Cancer. The unstandardized coefficients indicate the magnitude and direction of the relationship between each predictor and the dependent variable. Notably, Health Literacy emerges as a significant predictor (B = 0.148, *p* < 0.001), with a standardized coefficient (ß) of 0.449. This suggests that for every one-unit increase in Health Literacy, Knowledge about Cervical Cancer is expected to increase by 0.449 units. However, Perceived Susceptibility and Severity, Perceived Benefits, and Perceived Barriers do not significantly affect Knowledge about Cervical Cancer.

**Table 6:** Regression coefficients: health literacy and screening behaviors as predictors of knowledge about Cervical Cancer

Predictors	Knowledge about Cervical Cancer						
	Unstandardized Coefficients		Standardized Coefficients	t	***P***	95% CI for difference	
	***B***	***Std. Error***	***ß***			**Lower bound**	**Upper bound**
(Constant)	-0.534	0.796		-0.671	0.503	-2.099	1.031
Health Literacy	0.148	0.017	0.449	8.637	< 0.001	0.114	0.181
Perceived Susceptibility and Severity	0.030	0.032	0.051	0.927	0.355	-0.034	0.093
Perceived Benefits	0.054	0.044	0.071	1.240	0.216	-0.032	0.140
Perceived Barriers	-0.017	0.014	-0.059	-1.250	0.212	-0.045	0.010

Significant difference compared to the reference category ≤ 0.001

*ß* = standardized coefficient beta; *t =* Student t test for linear regression

95% CI for difference 95% Confidence Interval for Difference

*R*^*2*^ = 0.270 (*Adj R*^*2*^ = 0.261), *F-*change = 31.893, *P* ≤ 0.001

Dependent variable: Knowledge about Cervical Cancer

In Table 7, the regression coefficients are for Knowledge about Cervical Cancer and Screening Behaviors as predictors of Health Literacy. Knowledge about Cervical Cancer emerges as a significant positive predictor of HL (B = 1.205, *p* < 0.001), with a standardized coefficient (ß) of 0.396. Perceived Benefits also significantly predict Health Literacy (B = 0.579, *p* < 0.001), with a standardized coefficient 0.250. On the other hand, Perceived Barriers negatively predict HL (B=-0.124, *p* = 0.002), suggesting that higher perceived barriers are associated with lower health literacy levels.

**Table 7:** Regression coefficients: knowledge about Cervical Cancer and Screening behaviors as predictors of health literacy

Predictors	Health Literacy						
	Unstandardized Coefficients		Standardized Coefficients	t	***P***	95% CI for difference	
	***B***	***Std. Error***	***ß***			**Lower bound**	**Upper bound**
(Constant)	15.117	2.125		7.115	< 0.001	10.938	19.296
Knowledge	1.205	0.140	0.396	8.637	< 0.001	0.931	1.479
Perceived Susceptibility and Severity	0.124	0.092	0.069	1.353	0.177	-0.056	0.305
Perceived Benefits	0.579	0.121	0.250	4.786	< 0.001	0.341	0.817
Perceived Barriers	-0.124	0.039	-0.138	-3.142	0.002	-0.201	-0.046

Significant difference compared to the reference category ≤ 0.001

*ß* = standardized coefficient beta; *t =* Student t test for linear regression

95% CI for difference 95% Confidence Interval for Difference

*R*^*2*^ = 0.357 (*Adj R*^*2*^ = 0.350), *F-*change = 47.896, *P* ≤ 0.001

Dependent variable: Health Literacy

## Discussion

Cervical cancer significantly impacts women worldwide, with an exceptionally high burden in low- and middle-income countries [34]. According to a 2023 report, Egypt sees 1320 new cases of cervical cancer each year, leading to 744 deaths [35]. Health literacy is a critical factor in preventing this disease [21]. In 2023, Egypt’s Ministry of Social Solidarity launched a significant campaign called ‘Journey of a Thousand Kilometres’ to improve public awareness about cervical cancer and promote regular screenings. The campaign started in Alexandria and traveled through 11 cities, ending in Cairo [36]. A previous study (2022) conducted in Egypt found that knowledge and attitudes towards cervical cancer screening and HPV vaccination varied among healthcare professionals. About 45% had limited knowledge, and 57% had neutral to negative attitudes. Older, more experienced professionals were more likely to perform cervical cancer screenings, while younger, less experienced professionals were more likely to prescribe the HPV vaccine [37]. Despite these efforts, there is a significant knowledge gap about the link between health literacy and cervical cancer screening behaviors among Egyptian women. This study aimed to investigate this relationship to improve prevention strategies and enable women to make informed health decisions.

Most of our participants were unaware of CCS, with only 5 participants (1.4%) having undergone the screening. Additionally, only two participants reported receiving the HPV vaccine. These statistics align with the historically low rates of CCS in Egypt. A report from the (WHO., 2021) confirmed this, stating that less than 10% of Egyptian women had been screened for cervical cancer in the previous five years [38]. Several factors contribute to this situation. Firstly, comprehensive health education is deficient in Egypt, especially concerning cervical cancer prevention. Secondly, the incidence of cervical cancer in Egypt is relatively low, at a rate of 2.3 per 100,000 individuals per year, as documented by Arbyn et al. (2020) [2]. Lastly, the lack of a formal national program for cervical screening could be a significant contributing factor to the low awareness and screening rates. Al-Rifai & Loney (2017) used secondary data from the 2015 Egypt Health Issues Survey (EHIS) on a sample of 7,518 Egyptian women aged between 15 and 59. The study reported that a substantial 92.3% of the women reported a lack of knowledge about cervical smear cancer screening services [38, 39].

Consistent with our findings, Ghebrendrias et al. (2021) conducted a study involving 53 female refugees from Sub-Saharan Africa and the Middle East [7]. Their research revealed that more than half of the participants identified fear of pelvic examinations and concerns about modesty as barriers to accessing gynecological care, leading to almost 34% avoiding regular pap tests. Additionally, when questioned about the HPV vaccine, only one out of 18 participants was aware of it, and none had received it for themselves or their children. Interestingly, over 60% of the participants wanted to learn more about HPV and pap tests.

It is significant to note that a substantial number of the participants recognized that being a non-smoker reduces the risk of cervical cancer and the increased risk associated with having multiple sexual partners. This is consistent with the WHO (2023) report, which highlighted that smoking is a significant risk factor for cervical cancer [40]. Abnormal cells, which can eventually develop into cancer, can be caused by a persistent infection with high-risk HPV types. Several factors can influence this progression to cancer, including the oncogenicity level of the HPV type, the individual’s immune status, the presence of other sexually transmitted infections, the number of childbirths, the age at first pregnancy, the use of hormonal contraceptives, and smoking.

The current research revealed that a significant portion of the respondents firmly believed they would not be diagnosed with cervical cancer in the future, were not apprehensive about the potential risk, and disagreed that cervical cancer screening can reduce mortality. However, our participants’ lifestyle habits and patterns were quite revealing. A small percent reported regular exercise, while over half preferred less healthy dietary options such as fast food. A significant majority reported suffering from insomnia and being exposed to secondhand smoke. Alarmingly, only 5 participants (1.4%) reported having regular medical checkups. These findings align with a study by Aziz et al. (2022) involving a convenience sample of 235 premenopausal women seeking care at the obstetrics and gynecology departments of Minia University Maternity and Children Hospital. The study indicated that 36.3% of participants did not exhibit fear regarding the possibility of developing cervical cancer [41]. In contrast to our results, Aldohaian et al. (2019) discovered that 38% of participants recognized the severity of the disease, and 82% acknowledged the benefits of screening. However, 27% of participants identified barriers to undergoing a Pap smear test [42].

The current study identified time constraints, cost, and forgetfulness as perceived barriers to CCS among participants. Meanwhile, recommendations from close family members and healthcare providers influenced participation in CCS. These findings align with those of Salem et al. (2017), which involved female Saudi teachers [43]. In that study, an exploratory factor analysis revealed that personal fears, such as embarrassment from screening, were the primary hindrance to CCS. This was followed by factors related to healthcare organizations.

The data reveals a significant and positive correlation between knowledge of cervical cancer and health literacy among the participants. This finding aligns with a study by Saleem et al. (2019), which found that a majority of the 154 women with cervical cancer at Jimma University Teaching Hospital in Southwestern Ethiopia were illiterate, unaware of cervical cancer, and had an advanced stage of disease at diagnosis, given the low literacy rates and lack of knowledge about cervical cancer, which has been linked to reduced likelihood of screening [44]. Individuals with higher health literacy are more capable of seeking out, understanding, and retaining information about cervical cancer. They can understand the importance of screening and early detection and recognize the symptoms associated with cervical cancer. On a positive note, a substantial number of our participants demonstrated awareness of cervical cancer symptoms such as intermenstrual vaginal bleeding, persistent unpleasant discharge, and discomfort during intercourse. Awareness was also notable for postmenopausal bleeding and post-coital bleeding. However, despite this awareness and the understanding that Pap tests can identify cervical abnormalities before they progress to cancer, there is a clear need for further education to enhance knowledge in this area.

The study found a negative correlation between perceived barriers and knowledge about CC and HL. This aligns with research by Bazaz et al. (2019) involving 231 women from Iran, where factors like education, employment, income, advice from mothers and young friends, and study duration were significantly associated with health literacy [45]. The study also found a significant association between searching, study time, young friends’ counseling, and cervical cancer literacy scores. Another survey by Ebu et al. (2019) observed significant improvements in knowledge of cervical cancer, its screening, perceived seriousness, benefits, and barriers following an educational program [46]. The study concluded that health education interventions are essential for enhancing knowledge and perceptions, as well as boosting self-efficacy among women regarding cervical cancer and screening. These findings are consistent with a quasi-experimental study conducted on 65 women at an obstetric outpatient clinic at Benha University Hospital, Egypt. The study aimed to assess the impact of an educational intervention on women’s knowledge and attitudes regarding cervical cancer. Results showed that the mean knowledge score increased from 11.33 ± 7.28 pre-intervention to 21.20 ± 47 post-intervention. Before the intervention, none of the women exhibited a positive attitude, whereas after the intervention, 30.8% demonstrated a positive attitude [25].

It might be challenging for Egyptian women to schedule and attend medical appointments due to their work and family responsibilities. Additionally, the cost of medical screenings can be a significant barrier. Some women may not have the necessary insurance coverage or financial means to afford screening tests. Regular screenings are essential for early detection and treatment of cervical cancer. However, some women may forget to schedule or attend their appointments for various reasons, such as being preoccupied with other responsibilities or simply forgetting, and there is a lack of health education programs on CC either in MCH or through formal media coverage and social media. These barriers can hinder the effective management and prevention of cervical cancer. Therefore, addressing these issues is crucial for promoting health literacy and encouraging regular cervical cancer screenings.

### Implication for nursing practice

This study’s findings have profound implications for Egyptian women and future research. It emphasizes the urgent need for health education initiatives designed to increase cervical cancer awareness and the importance of its screening practices. By addressing misconceptions and enhancing knowledge through culturally appropriate and accessible means such as Primary Health Care Centers, Obstetric outpatient clinics, medical caravans for rural areas, TV programs, and social media, we could see an improvement in the utilization of cervical cancer screening services. The study also highlights the significance of lifestyle factors, such as sleep patterns and exposure to secondhand smoke, on overall health. The study further highlights the necessity for interdisciplinary collaboration in healthcare. Nurses and other healthcare professionals can develop a holistic approach to women’s health by working together.

From a research perspective, this study contributes valuable insights into the complex interplay of sociodemographic factors, lifestyle behaviors, health literacy, and cervical cancer screening practices among Egyptian women. Future research in this area could delve deeper into understanding the barriers that hinder women from accessing screening services and develop targeted interventions to address these challenges. Additionally, exploring the role of social determinants of health, such as access to healthcare and socioeconomic status, could provide further insights into the factors influencing women’s health-seeking behaviors. Overall, this study lays a foundation for future research to continue exploring ways to improve cervical cancer prevention and control efforts among Egyptian women.

### Limitations

Our study examining the relationship between health literacy and cervical cancer screening behaviors among Egyptian women is subject to several limitations that warrant consideration. Firstly, the reliance on self-reported data for health literacy and screening behaviors poses a risk of response bias. Participants may have inaccurately reported their literacy levels or screening practices, leading to skewed results. Additionally, the cross-sectional design of our study allows us to identify associations between variables but precludes us from establishing causality. While we may observe a correlation between health literacy and screening behaviors, we cannot definitively conclude that one factor directly influences the other. Furthermore, the tools used to measure health literacy and screening behaviors have yet to be culturally validated for the Egyptian population, raising concerns about measurement validity. With culturally appropriate instruments, the accuracy and reliability of our findings may be maintained. Moreover, our study did not comprehensively address factors such as fear of the screening procedure, access to healthcare services, or cultural stigmas surrounding cancer, potentially confounding our results. Despite these limitations, our study provides valuable insights into the complex interplay between health literacy and cervical cancer screening behaviors among Egyptian women, highlighting areas for further investigation and intervention.

## Conclusion

The study provides a detailed examination of socio-demographic characteristics, lifestyle behaviors, cervical cancer screening (CCS) practices, awareness of cervical cancer (CC), and health literacy (HL) among Egyptian women. The sample is diverse, with varying age groups, educational backgrounds, and marital statuses, predominantly residing in urban areas and nuclear families. Despite high levels of education, a significant proportion of participants perceive their income as insufficient. Lifestyle behaviors such as irregular sleep patterns and passive smoking exposure are prevalent, while engagement in regular exercise and healthy dietary habits is low. CCS uptake is notably low, with most participants reporting not hearing about CCS or undergoing screening. Awareness of CC symptoms and risk factors is relatively high, yet knowledge gaps persist, particularly regarding the role of Pap tests in detecting cervical abnormalities. Perceived barriers to screening include time constraints, cost concerns, and forgetfulness. The study highlights the importance of health literacy in improving knowledge about CC, with higher HL associated with increased knowledge. Perceived benefits of CCS and perceived barriers to HL significantly predict HL levels, indicating the complex interplay of factors influencing health behaviors and literacy among Egyptian women.

## Data Availability

The datasets generated and analyzed during the current study are not publicly available due to confidentiality agreements but are available upon reasonable request from the corresponding author.
